# Land cover changes induced by the great east Japan earthquake in 2011

**DOI:** 10.1038/srep45769

**Published:** 2017-03-31

**Authors:** Mitsunori Ishihara, Takeo Tadono

**Affiliations:** 1 Japan Aerospace Exploration Agency, 2-1-1 Sengen, Tsukuba, 305-8505, Japan

## Abstract

The east Japan earthquake that occurred on March 11, 2011 was a big natural disaster, comprising the large earthquake shock, tsunami, and Fukushima Daiichi Nuclear Power Plant (FDNPP) accident. These disasters caused changes in the land use and land cover (LULC) in Japan’s Tohoku district. While the LULC map created before the disaster is available, as yet there is no precise LULC map of the district after the disaster. In this study, we created a precise LULC map for the years 2013–2015 post-disaster with 30-m spatial resolution using the Landsat-8 with the Operational Land Imager (OLI) to evaluate the changes in LULC induced by the disaster. Our results indicate many changes in areas categorized as rice paddies primarily into grass categories along the coast damaged by the tsunami and in the evacuation zone around the FDNPP. Since there is a possibility of future LULC changes according to the change of the evacuation zone and implementation of reconstruction and revitalization efforts, we recommend continual monitoring of the changes in LULC by the use of satellite data in order to evaluate the long-term effects of the disaster.

The east Japan earthquake of March 11, 2011 caused great damage in the northeast region of Japan. This earthquake triggered three catastrophes i.e., the shock of the earthquake, a tsunami, and the FDNPP accident. While these disasters had each occurred separately in times past, e.g., the Indian Ocean tsunami caused by the Sumatra-Andaman earthquake and the Chernobyl nuclear disaster^1^,^2^, their simultaneous occurrence had not. The earthquake occurred with the magnitude 9.0 and was one of the most devastating natural disasters in past decades^3^. The immense tsunami induced by the earthquake affected a 2,000-km stretch of Japan’s Pacific coast and inundated over 400 km^2^ of land^4^. As of September 2016, the official fatality count for the tsunami disaster totaled 15,894 people with an additional 2,557 people missing^5^. Furthermore, the FDNPP was damaged by the tsunami and released large amount of radioactive materials into the atmosphere^6^,^7^. These radioactive materials extended from the FDNPP to the northwest, and this area was subsequently designated as the evacuation zone by the Japanese government. In April 2016, this evacuation zone encompassed an area of 953 km^2^, and by June 2015 about 79,000 people had been evacuated from that zone^8^,^9^.

These disasters have had multiple effects on various ecosystems and human activities. For example, agricultural activity has stagnated due to the saltwater damage caused by the tsunami^10^,^11^. Furthermore, abandoned farmlands and wildlife numbers have increased in the evacuation zone and surrounding areas where human activities are restricted due to the direct or indirect effects of the FDNPP accident^12^. With respect to reconstruction and disaster prevention efforts, because the effects of the tsunami and the FDNPP accident may expand further in several ecosystems, it is important to accurately assess and quantify these effects.

With respect to the assessment process, LULC maps can be used in the numerical simulation of the dispersion process of radioactive materials, the formulation and implementation of reconstruction and revitalization efforts, and the creation of hazard maps^13^,^14^. Because the disaster areas of the tsunami and the nuclear accident are widespread, the use of satellite data to construct an LULC map is reasonable. A precise LCLU map for all of Japan for the years 2006–2011 was provided by the Japan Aerospace Exploration Agency (JAXA, http://www.eorc.jaxa.jp/ALOS/lulc/jlulc_jpn.htm). The data were obtained at 10-m spatial resolution by the use of multi-temporal images of the Advanced Visible and Near Infrared Radiometer type-2 (AVNIR-2) onboard the Advanced Land Observing Satellite (ALOS). This satellite data product has generated the highest-spatial-resolution LULC map for all of Japan, and has been used in several researches^15^,^16^,^17^,^18^,^19^. Unfortunately, the operation of the ALOS AVNIR-2 officially ended in May 2011, so no recent LULC map has been created since the great east Japan earthquake.

On the other hand, previously in this area, changes in LULC have been assessed to create new maps post-disaster using changes of several vegetation indices calculated by multi-temporal low spatial resolution data from the Moderate Resolution Imaging Spectroradiometer (MODIS)^3^,^15^. However, it is difficult to assess precise changes of LULC for disaster-damaged areas because a spatial resolution of MODIS data is 250 m and the land cover within the area is highly heterogeneous^15^. Therefore, to accurately assess the LULC effects of the disaster, we must create a precise post-disaster LULC map. As such, we produced our precise LULC map for the years 2013–2015 post-disaster with a 30-m spatial resolution using Landsat-8 with the OLI. To evaluate the changes in LULC due to the great east Japan earthquake, we compared the LULC maps before and after the disaster.

## Results and Discussion

**Figure 1:**
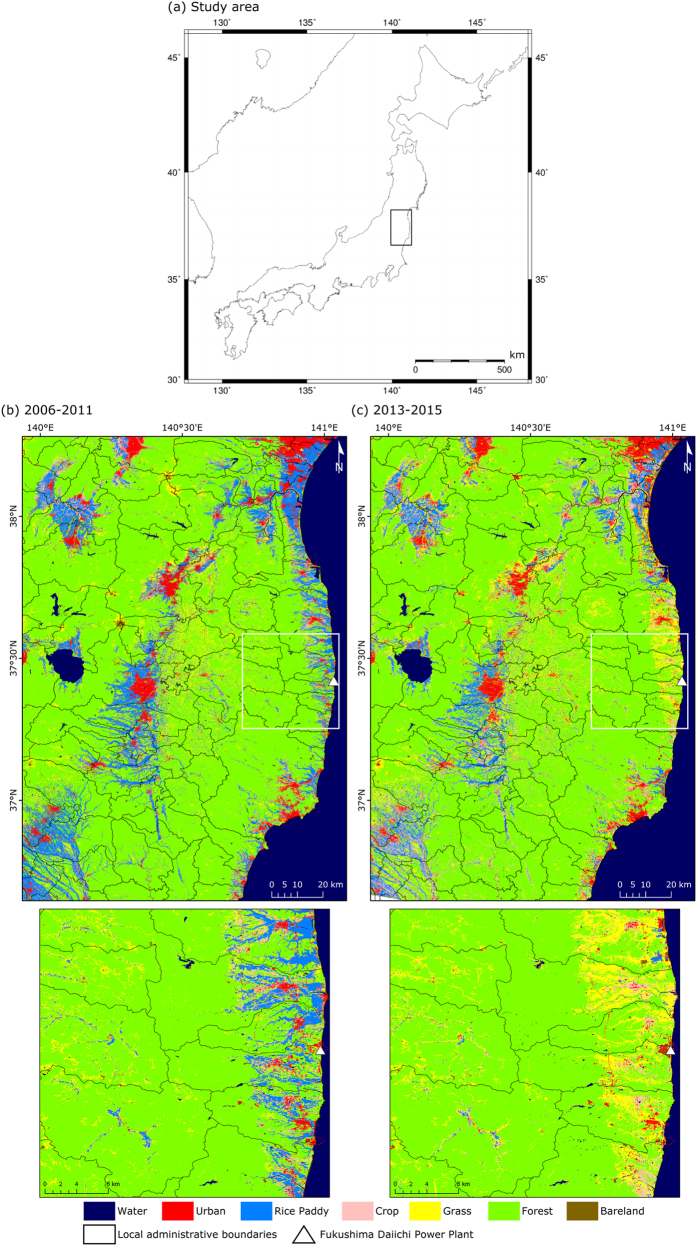
The LULC maps in the study area. The study area (**a**) and LULC map produced by ALOS AVNIR-2 in the years 2006–2011 (**b**) and Landsat-8 OLI in the years 2013–2015 (**c**). (**a**) was created with the Generic Mapping Tools (GMT) 5.1.2 (http://gmt.soest.hawaii.edu/). (**b**) was downloaded from the JAXA (http://www.eorc.jaxa.jp/ALOS/lulc/jlulc_jpn.htm). (**c**) was created using the Landsat-8 OLI surface reflectance product from the USGS EarthExplorer (http://earthexplorer.usgs.gov/) and processed using own classification program^20^,^21^ as detailed in the methods section.

Figure 1 shows the LULC maps produced by the ALOS AVNIR-2 for the years 2006–2011 (Fig. 1b) and the Landsat-8 OLI for the years 2013–2015 (Fig. 1c). The LULC maps based on ALOS AVNIR-2 data was provided by JAXA. These maps were created with a kernel-based machine-learning classification using time-series multi-temporal optical images^20^,^21^, and the ALOS AVNIR-2 LULC map also used other ancillary data (methods). The LULC categories are defined as Table 1. The overall accuracies of the LULC maps categorized 8 LULC types (water, urban, rice paddy, crop, grass, forest, and bareland) by the LANDSAT-8 OLI and ALOS AVNIR-2 are 85.1% and 85.8%, respectively and the kappa coefficient are 0.799 and 0.824, respectively (Table 2). Before the disaster, large areas categorized as rice paddies were spread throughout the lowland along the coast. After the disaster, these rice paddies have since become categorized mainly as grass. These changes occurred after the disaster because rice cropping had not been performed due to tsunami damage. The spatial distribution of the changes in the rice paddy category is similar to the spatial distribution of the tsunami-damaged area^22^,^23^. Furthermore, because it is difficult to continue agricultural activities in the evacuation zone and the high-radioactive contamination area, many agricultural lands in that area were abandoned. In the high-radioactive contamination area, there is a high possibility of exceeding the standard radiation dose threshold for agricultural products^24^,^25^,^26^. A previous study reported similar results of detected significant vegetation changes using MODIS data in most rice paddy categories within the 20-km-radius evacuation zone around the FDNPP^15^.

**Figure 2:**
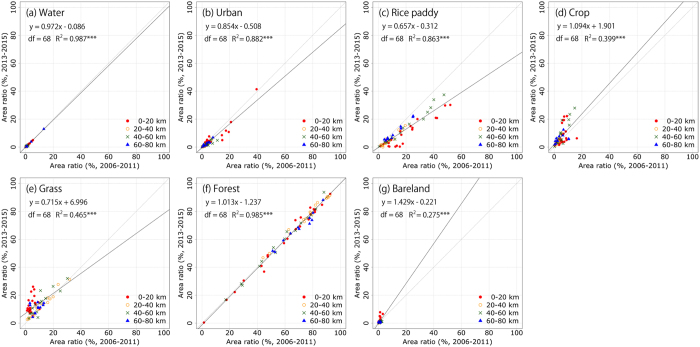
The comparison of area ratio of the LULC map categories for each municipality in 2006–2011 and 2013–2015. (a–g) The water, urban, rice paddy, crop, grass, forest, and bareland categories, respectively. Points in these figures are categorized municipalities into four zones of 0–20, 20–40, 40–60, and 60–80 km by the difference of the distance from the coastline to center of municipality. The solid gray line shows the 1:1 relationship. The solid black line shows the linear regression line. df and R^2^ shows the degrees of freedom and coefficient of determination, respectively. ***p < 0.001.

Next, we compared the area ratio of the LULC categories for each municipality in the 2006–2011 and 2013–2015 periods (Fig. 2). The area ratio was calculated by dividing the area of each LULC category in municipality by the area of municipality. We categorized the municipalities into four zones of 0–20, 20–40, 40–60, and 60–80 km with respect to the distance from the coastline to the municipality center to evaluate the effects of the tsunami. In the water and forest categories, almost all the data in the scatter plots were close to the 1:1 ratio in all distance zones (Fig. 2a and f). In the rice paddy category, however, the area ratio showed a decreasing trend after the disaster, with some points almost 0% in the 0–20-km zone (Fig. 2c). Along with the decreasing area ratio in the rice paddy category, the area ratio in the grass category increased after the disaster (Fig. 2e). Furthermore, these large decreasing and increasing trends after the disaster in each category occurred mainly in the 0–20-km zone. This is because several types of weed that are categorized as grass expanded into the abandoned rice paddy fields following the tsunami and FDNPP accident, and these areas have not been maintained since the disaster^27^,^28^.

The area ratios in the urban and bare land categories show slightly decreasing and increasing trends after the disaster, respectively (Fig. 2b and g). These post-disaster changes also mainly occurred in the 0–20-km zone near the coastline. In the coastal region, areas heavily damaged by the tsunami became vacant lots because many buildings were washed away. Although these buildings have been under phased reconstruction, there remain many vacant lots in this area. In contrast, the area ratio for the crop category shows both increasing and decreasing trends after the disaster (Fig. 2d). While there are some places that can no longer be cultivated due to the effects of the tsunami or radioactive materials, there is the possibility of cultivation in other areas.

**Figure 3:**
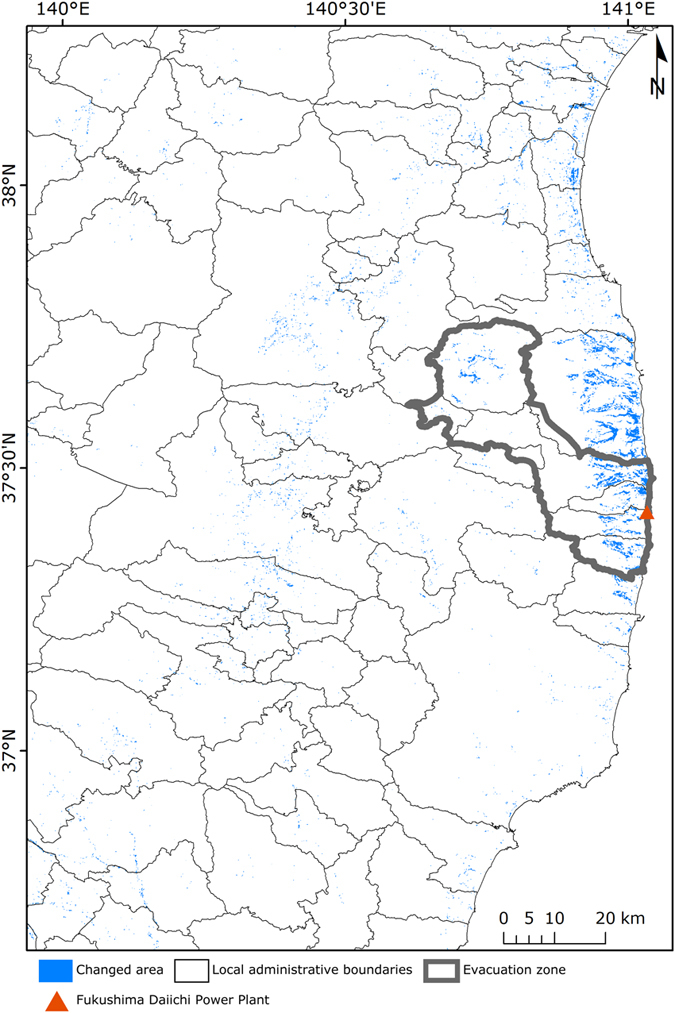
The spatial distribution of the area where changed from the rice paddy category in the years 2006–2011 into the grass category in the years 2013–2015. Map was created with GRASS GIS version 7.0 and ArcMap version 10.2.2 (https://www.arcgis.com/).

We extracted the areas that changed categories from rice paddy in the years 2006–2011 to grass in the years 2013–2015 in order to examine the distribution of the main changes following the disaster (Fig. 3). We detected large changes over a wide range in the coastal region and evacuation zone, and therefore consider that the rice paddy category changed mainly into the grass category by effects of the tsunami and nuclear accident. Slight changes also occurred in the rice paddy category in places other than the coastal region and evacuation zone. However, it is difficult to determine whether these changes are a direct influence of the disaster because there is a possibility that differences in the accuracies and spatial resolutions of the LULC maps may have influenced these changes.

The LULC in the disaster-affected area changed drastically in a short period of time due to effects of the tsunami and nuclear accident. Because we have little experience with drastic widespread changes in LULC, we must examine how these changes influence the surrounding environment. After the disaster, many researchers used a variety of simulation models to simulate the diffusion or migration of radioactive materials, such as ^131^I and ^137^Cs^18^,^19^,^29^,^30^. These simulations used LULC map information as input data, and there is a possibility that the calculation results were influenced by changing ground surface conditions. For example, Kinase *et al*.^18^ reported that the ecological half-life of radioactive cesium changed depending on the LULC, and this value in forest areas was much larger than those in other LULC areas. These simulations have almost all used LULC maps produced before the disaster, despite the fact that it is important to use current LULC map information to generate future predictions.

There is a high possibility that LULC will change in the future. In particular, this is so because human activities, e.g., agricultural activities, have been restricted in the evacuation zone and high-radioactive contamination area, so abandoned farmlands have become grasslands and are anticipated to ultimately change into forest^27^,^28^. The effects of radiation are predicted to continue for a long time, so it is important to continually monitor changes in LULC. Satellite observation is readily available for continual monitoring over wider areas and our study results suggest the potential of satellite monitoring. These continual data can be utilized in basic and applied research with respect to earthquake disaster reconstruction and in the practical formulation and implementation of reconstruction and revitalization plans by governmental agencies.

## Methods

### Satellite data

To create the LULC map, we downloaded the Landsat-8 OLI surface reflectance product from the United States Geological Survey (USGS) EarthExplorer, which is an online data provisioning service, for the period from 2013 to 2015^31^. We used 45 images that covered study area (path 107/row 034) except for 100% cloud-covered images (Supplementary Table S1). In our preprocessing scheme, we generated subsets of our study region from all of the images (Fig. 1b) with Geospatial Data Abstraction Library (GDAL) version 1.11.3 (http://www.gdal.org/) and masked cloud, cloud shadow, and snow pixels using the C version of the Function of Mask (CFmask) band^32^ with Geographic Resources Analysis Support System (GRASS) GIS version 7.0 (https://grass.osgeo.org/).

### Training and validation data

We collected 800 training data points from Google Earth and Street View by visual assessment. We checked the satellite data of Google Earth and the images of Google Street View for the period from 2013 to 2015 and selected 100 points from each LULC category (water, urban, rice paddy, crop, grass, deciduous forest, evergreen forest, and bare land) for homogeneous areas more than 30-m in diameter. We could collect appropriate training data from Google Earth and Street View because these selected categories are typical Japanese LULC and we have collected many ground based reference data for LULC maps around the study area.

We collected validation data from the “Site-based dataset for Assessment of Changing Land cover by JAXA (SACLAJ).” This database is maintained for the collection of reference data for LULC maps in the ground based measurement and has stored over 46,000 points mainly in Japan. Each point contains information about its geolocation, observation date, LULC category, and homogeneity as well as a photo. In this study, we used 564 points in the study area within the period from 2013 to 2015.

### Classification and change detection

We created the LULC map using a kernel-based probabilistic classification (KPC), which is based on Bayesian inference^20^,^21^. This is a probabilistic model that represents a distribution of observable data, given some hidden parameters. KPC constructs a generative model from training data using kernel density estimation, which is a non-parametric method for estimating probability density. This model, a detailed description of which was made by Hashimoto *et al*.^17^, calculates the joint probability from the multi-temporal posterior probability of each class and then normalizes the joint probability distribution, such that the joint probabilities of all classes add up to 1. Finally, the LULC class with the highest joint probability of all the LULC classes is selected as the classification result.

We categorized 8 LULC types (Table 1) using the reflectances from band 1 to band 7 of the Landsat-8 OLI and the training data. In the LULC map of the Landsat-8 OLI, we merged the deciduous and evergreen forest categories into the forest category, since there is only a low possibility that the forest type changed in a few years. In the LULC map by the ALOS AVNIR-2, 10 LULC types (Table 1) were categorized by JAXA using all reflectances of the ALOS AVNIR-2 band. This map was used ancillary data such as ALOS Phased Array type L-band Synthetic Aperture Radar (PALSAR) for water body and Suomi National Polar-orbiting Partnership (Suomi NPP) night lights data for urban area. We also merged the deciduous broadleaf forest, deciduous needleleaf forest, evergreen broadleaf forest, and evergreen needleleaf forest categories into the forest category.

We resampled these maps to a 90-m spatial resolution in order to avoid the influence of geolocation error and differences in spatial resolution between the ALOS AVNIR-2 and Landsat-8 OLI using a mode-resampling method, which selects the value that appears most often of all the sampled points with GDAL version 1.11.3. After that we compared these LULC maps to extract the areas that changed categories after the disaster.

## Additional Information

**How to cite this article**: Ishihara, M. and Tadono, T. Land cover changes induced by the great east Japan earthquake in 2011. *Sci. Rep.* **7**, 45769; doi: 10.1038/srep45769 (2017).

**Publisher's note:** Springer Nature remains neutral with regard to jurisdictional claims in published maps and institutional affiliations.

## Supplementary Material

Supplementary Information

## Figures and Tables

**Table 1:** The definition of LULC map categories based on the International Geosphere-Biosphere Programme (IGBP)^33^ and Global Land Cover by National Mapping Organizations (GLCNMO)^34^ land cover units.

Landsat-8 OLI	ALOS AVNIR-2	LULC class definition
Water	Water	Oceans, seas, lakes, reservoirs, and rivers. Can be either fresh or saltwater bodies.
Urban	Urban	Land covered by buildings and other man-made structures.
Rice paddy	Rice paddy	The cover type is rice paddy influenced by the presence of water.
Crop	Crop	Lands covered with temporary crops followed by harvest and a bare soil period (e.g., single and multiple cropping systems). Note that perennial woody crops will be classified as the appropriate forest or shrub land cover type.
Grass	Grass	Lands with herbaceous types of cover. Tree and shrub cover is less than 10%.
Deciduous Forests	Deciduous Broadleaf Forests	Lands dominated by woody vegetation with a percent cover >60% and height exceeding 2 m. Consists of broadleaf tree communities with an annual cycle of leaf-on and leaf-off periods.
	Deciduous Needleleaf Forests	Lands dominated by woody vegetation with a percent cover >60% and height exceeding 2 m. Consists of seasonal needleleaf tree communities with an annual cycle of leaf-on and leaf-off periods.
Evergreen Forests	Evergreen Broadleaf Forests	Lands dominated by broadleaf woody vegetation with a percent cover >60% and height exceeding 2 m. Almost all trees and shrubs remain green year round. Canopy is never without green foliage.
	Evergreen Needleleaf Forests	Lands dominated by needleleaf woody vegetation with a percent cover >60% and height exceeding 2 m. Almost all trees remain green all year. Canopy is never without green foliage.
Bareland	Bareland	Lands with exposed soil, sand, rocks, or snow and never has more than 10% vegetated cover during any time of the year.

**Table 2:** Confusion matrix of the LULC map produced by Landsat-8 OLI in the years 2013–2015 (a) and ALOS AVNIR-2 in the years 2006–2011 (b).

	Validation									
		Water	Urban	Rice paddy	Crop	Grass	Forest	Bareland	Total	Producer’s accuracy (%)
**(a) Landsat-8 OLI**										
Results	Water	14	0	1	2	0	0	0	17	82.4
	Urban	0	42	0	4	0	0	2	48	87.5
	Rice paddy	0	1	146	2	2	0	0	151	96.7
	Crop	0	2	8	26	15	1	2	54	48.1
	Grass	0	3	4	7	35	9	1	59	59.3
	Forest	0	0	1	1	5	214	0	221	96.8
	Bareland	0	9	0	2	0	0	3	14	21.4
	Total	14	57	160	44	57	224	8	564	—
	User’s accuracy (%)	100	73.7	91.3	59.1	61.4	95.5	37.5	—	85.1
	Kappa coefficient: 0.7999									
**(b) ALOS AVNIR-2**										
Results	Water	193	1	1	0	0	0	2	197	98.0
	Urban	2	222	2	1	0	0	2	229	96.9
	Rice paddy	1	2	260	18	6	3	1	291	89.3
	Crop	1	2	28	76	41	15	5	168	45.2
	Grass	0	0	10	14	42	14	1	81	51.9
	Forest	1	1	12	9	7	394	0	441	95.2
	Bareland	0	2	1	1	3	0	22	29	75.9
	Total	198	230	304	119	99	426	33	1409	—
	User’s accuracy (%)	97.5	96.5	85.5	63.9	42.4	92.5	66.7	—	85.8
	Kappa coefficient: 0.824									

The result of ALOS AVNIR-2 was provided by the JAXA (http://www.eorc.jaxa.jp/ALOS/lulc/jlulc_jpn.htm). Forest category was merged the deciduous and evergreen forest categories of Landsat-8 OLI and the deciduous broadleaf forest, deciduous needleleaf forest, evergreen broadleaf forest, and evergreen needleleaf forest categories of ALOS AVNIR-2.
