# Supplementary material for: Land cover changes induced by the great east Japan earthquake in 2011
Source: Sci Rep. 2017 Mar 31;7:45769. doi: 10.1038/srep45769 (PMC5374709; doi:10.1038/srep45769)
Supplement: Supplementary Information [file srep45769-s1.pdf]

## Supplementary information

Land cover changes induced by the great east Japan earthquake in 2011

Mitsunori Ishihara[1*] and Takeo Tadono[1]

[1] Japan Aerospace Exploration Agency, 2-1-1 Sengen, Tsukuba, 305-8505, Japan

[*]Corresponding author

Mitsunori Ishihara

E-mail: ishihara.mitsunori@jaxa.jp

Tel: +81-50-3362-7444

**Supplementary Table S1| The lists of acquisition date of Landsat-8 OLI for this study.**

| 2013 | 2014 | 2015 |
|---|---|---|
| 2013.04.26 | 2014.01.07 | 2015.01.10 |
| 2013.05.12 | 2014.01.23 | 2015.02.27 |
| 2013.06.13 | 2014.02.24 | 2015.03.15 |
| 2013.06.29 | 2014.03.12 | 2015.03.31 |
| 2013.07.15 | 2014.03.28 | 2015.04.16 |
| 2013.08.16 | 2014.04.13 | 2015.05.02 |
| 2013.09.01 | 2014.04.29 | 2015.05.18 |
| 2013.09.17 | 2014.05.31 | 2015.07.21 |
| 2013.10.03 | 2014.06.16 | 2015.08.06 |
| 2013.11.20 | 2014.07.02 | 2015.08.22 |
| 2013.12.06 | 2014.08.03 | 2015.09.23 |
| 2013.12.22 | 2014.08.19 | 2015.10.09 |
| | 2014.09.04 | 2015.10.25 |
| | 2014.09.20 | 2015.12.12 |
| | 2014.11.07 | 2015.12.28 |
| | 2014.11.23 | |
| | 2014.12.09 | |
| | 2014.12.25 | |
